# A bibliometric analysis of circular economy in the fields of business and economics: towards more action-oriented research

**DOI:** 10.1007/s10668-022-02347-x

**Published:** 2022-04-30

**Authors:** Miha Dominko, Kaja Primc, Renata Slabe-Erker, Barbara Kalar

**Affiliations:** Institute for Economic Research, Kardeljeva ploščad 17, 1000 Ljubljana, Slovenia

**Keywords:** Circular economy, Sustainability, Closed-loop system, Bibliometric analysis, Future research

## Abstract

In this paper, we reveal and systemize development trends in the scientific field of the circular economy (CE). Our results imply that academic research on the CE focuses heavily on theoretical conceptualizations and technological solutions. However, these advancements alone are unlikely to help prevent from ecological collapse. With this observation in mind, we explore the potential held by a more relational, action-based approach to support a faster and more efficient transition from the linear economy to the CE. A useful combination of several bibliometric techniques gave us valuable references for re-focusing this area of science to adopt action-oriented research where a group of stakeholders collaborates and co-creates solutions. An analysis of valuable action-oriented CE studies reveals that scholars focus on the techno-economic aspect, where they develop ways to create optimal circular material and energy flows and co-design processes for products/services, as well as the organizational aspect, where they study self-sustainable community networks and participatory governance. We identify three research streams that would benefit from such action-oriented research for a faster practical implementation: sustainable supply chains, waste management, and business model innovation. A practice-based agenda is proposed to stimulate the scientific community to conduct future research on a CE that better supports companies.

## Background

Today’s patterns of production and consumption pose a serious threat to the Earth’s biocapacity (Wackernagel & Beyers, 2019). Increases in the world’s population, urbanization, and modernization mean that energy consumption is growing rapidly, driving up greenhouse gas emissions and impacting the global climate (Lipson et al., 2019). Further critical issues are the geological scarcity of critical raw materials (Henckens et al., 2016; Massari & Ruberti, 2013) and the large amounts of waste being generated. Particularly worrying is the accumulation of mismanaged plastic waste (Lebreton & Andrady, 2019). These and many other environmental issues related to modern economic practices have stimulated interest in aligning global industrial systems with natural equilibria (Borrello et al., 2020). If we are to meet the UN’s global sustainable development goals (2015) and ensure consumption and production patterns that are more sustainable, the adoption and implementation of circular economy (hereafter CE) practices are urgently needed. Circularity has been put forward as a key factor in the search for long-term sustainability and for supporting it by way of growth in GDP, new jobs, and resource productivity (EC, 2014). However, to bring about the all-important changes in the provision of social justice, equity, and inclusion along with consumer lifestyles (Jaeger-Erben et al., 2021), all actors in society must work together and be committed. The Ellen McArthur Foundation is playing a major role in the CE’s expansion and has developed several practical initiatives governments around the world have followed. Although CE is a practitioner-driven concept, in this article we are interested in how science can support its faster implementation in practice, given the obstacles slowing its progress down that are mostly cultural in origin, particularly those regarding consumers and company culture (Kirchherr et al., 2018).

The essence of a circular system is to keep the value of resources, materials, and products in the economy for as long as possible (Merli et al., 2018). The CE may be seen as an integrated framework in which numerous concepts, such as industrial symbiosis, industrial ecology, life cycle assessment, cradle to cradle, biomimicry, and others, are explored. The desire to better understand the CE is reflected in the number of review studies. Most are narrowly focused on diverse, albeit related aspects of CE like industrial ecology (Saavedra et al., 2018), industrial symbiosis (Mallawaarachchi et al., 2020; Turken & Geda, 2020), Chinese public policy (Cui & Zhang, 2018), circular bioeconomy (e.g. Gregg et al., 2020), and resource conservation and recycling (Ji et al., 2018). However, one can also find review studies that cover the concept more comprehensively (e.g. Goyal et al., 2021; Homrich et al., 2018; Khitous et al., 2020; Mas-Tur et al., 2019; Merli et al., 2018). Specifically, in economics and business, two types of CE review studies dominate those dealing with supply-chain management (Govindan & Hasanagic, 2018; Lahane et al., 2020; Lis et al., 2020; Liu et al., 2018) and those dealing with business models (Ferasso et al., 2020; Ferreira Gregorio et al., 2018; Lahti et al., 2018; Lüdeke‐Freund et al., 2019). While these studies vary in their theoretical frameworks and research methods, they share the struggle to assess the actual state of the practice of adopting the CE in companies (Colzalari et al., 2021).

The literature on the CE has a rich tradition within scientific research, focusing on the development of methods and tools to support companies in their transition. Still, it largely remains conceptual and the informal process of how routines and guidelines are developed into formal CE practices is still a black box (Bocken et al., 2019; Pieroni et al., 2020). An obvious reason is that implementing CE practices requires the company to transform the way it creates, delivers, and captures value (Frishammar & Parida, 2019). It is argued here that the successful implementation of CE practices relies on the involvement of key stakeholders (Brown et al., 2019). The process of discussing routines and guidelines, which can be translated into assessments of smart solutions by the parties involved, is necessary (Balau et al., 2020). The argument for action-oriented research to co-produce solutions and translate visions into actions has been proposed by numerous CE scholars and practitioners (Valenturf & Purnell, 2021), although this approach requires skills and leadership to organize and manage the process (Meijerink & Bondarouk, 2018). Turning to the main terms used in the CE literature, only 2% of scientific contributions speak of participation, one of the vital components of the CE (Valenturf & Purnell, 2021).

There are certainly many instances in which popular quantitative predictions may be helpful. Forecasts of sales, employee turnover, and growth rates are illustrative. Yet, not all forms of psychosocial inquiry share the intention to predict and control. Specifically, according to Gergen (2014), action-oriented research, first introduced by Kurt Lewin (1946) to study organizational development, differs greatly from traditional empiricism in one respect. While the empiricists’ assumptions are based on a world of stable structures and processes in which knowledge can be accumulated over time, in contrast, action-oriented research builds on the premise of the volatility, complexity, and uncertainty of the environment. The challenge of an action-oriented researcher is to change the world instantly in collaboration with individuals, groups, and/or organizations by showing others how they might proceed. Attempts to increase knowledge through repeated studies, in such a researcher’s vision, are problematic (Gergen, 2014). These researchers might vary in their desire to understand others’ experiences, increase social justice, directly affect social and environmental change, and so on.

Action-oriented research aims to improve strategies, practices, and knowledge by combining research and practical work. It focuses on understanding organizational problems within specific socio-cultural contexts and the practice of problem-solving built on recurring cycles of action and reflection (Dickens & Watkins, 1999). The research process has three integrated aspects: participation, action, and research. The concentration on dialogue and actions among different stakeholders promotes collaboration but can also add insights into new organizational/community transformations. The ultimate result is the creation of contextual knowledge according to the needs of the subjects in focus (Gergen, 2014; Morales, 2016). Given these distinct research values, we believe that it is a promising approach for the CE field.

The present study aims to fill the gap in previous research by exploring the evolutionary trends of the CE and its co-existence with action-oriented research. This will answer the following question: How can a context-related approach, whereby the researcher focuses on the unique contextual features and an evaluation of their consequences, support the faster and more efficient transition from the linear economy to the CE? We first provide a systematic bibliometric analysis of CE research in the fields of business and economics, which we complement with a qualitative literature review of action-oriented research on the CE. By employing a two-step approach and combining several bibliometric techniques, we are able to provide an evolutionary overview of the research field, which ultimately allows us to explore how future research on the CE can better support companies and expand the discussion by considering the prospects of moving the concept from theory to practice by gaining the insights of action-oriented research. While individual techniques have their own strengths and weaknesses, the multi-faceted research methodology applied gives a more objective assessment of past, current, and future progress in the field.

## Step 1: A bibliometric analysis of CE research in the fields of business and economics

We begin Step 1 by presenting our data collection process. We then provide a thorough overview of the bibliometric methods used. We continue by providing a detailed description of our dataset, as well as the results of our analyses. The results are structured in three parts (past, present, future), which allows us to identify the antecedents, current studies as well as future trends in CE research in the business and economics fields.

Bibliometric methods are driven by different citation analyses of primary and secondary publications. While primary publications are identified using keywords, secondary publications are those that are cited by the identified primary publications. To create a broad dataset of primary publications on CE in the fields of business and economics, we used the Web of Science Core Collection, which contains all the necessary article and citation data. We started our topic search with the search term “circular economy”. Using the appropriate Boolean operators (AND and OR), we then limited our search to articles that focus primarily on the CE in business and economics by including the following additional topic searches: “business”, “economics”, “management”, and “organisation”. Finally, we limited the search to research articles in English (see footnote 1 for the precise search notation).^1^ The presented approach returned 4627 articles, which are our primary publications. These cite 212,064 documents, which are our secondary publications. The sample was obtained in January 2022.

To check the robustness of the dataset obtained, we ran extra search queries, where we included additional search words. First, we wanted to check whether synonyms for the CE, specifically “closed-loop economy” and “zero waste economy”, have been used in different research contexts and are hence not appropriately represented in our database. Since the search query revealed that the number of search results expands by only a handful of articles, we were able to conclude that these terms are generally treated as synonyms in the literature. We then repeated the exercise to test whether we had restrained our database to such an extent that essential articles on CE in the fields of business and economics were left out. Upon including keywords such as “recycling”, “reuse”, and “remanufacturing”, the number of articles increased significantly. However, a review of titles revealed that the vast majority of these articles did not relate to CE research in the fields of business and economics. Finally, because our database of articles is relatively small, we qualitatively reviewed whether it contained the most relevant articles while not broadening it to such an extent that would seriously affect the results. The review revealed that we can be confident in the database obtained.

Bibliometric methods have seen a strong resurgence recently due to the increased availability of online databases providing article and citation data, as well as the development of new and improved analysis software (Župić & Čater, 2015). They are based on cited references, which may be viewed as a representation of the publication itself as well as a symbol for diverse methodologies, data types, theoretical statements, etc. (Kullenberg & Nelhans, 2015; Small, 1978). Citations are also an expression of the publication’s importance. Since scholars refer to older publications to support their notions, the total sum of citations is the most crucial measure of a publication’s significance for a field of knowledge (Garfield, 1979; Small, 1978). In our analysis, we use two bibliometric methods: co-citation analysis (Small, 1973) as well as bibliographic coupling (Kessler, 1963). Each examines citation-based relationships between primary and secondary publications in its own way, complementing each other and allowing us to answer several research questions regarding the past, present, and future of CE research in the fields of business and economics. In Table 1, we provide a short overview of the two methods, including their focal points, methodological mechanisms, time frames, and sensitivity, as well as their advantages and limitations.

**Table 1:** Co-citation analysis and bibliographic coupling

	Co-citation analysis	Bibliographic coupling
Focal point	Cited (secondary) publications that are cited together in the citing (primary) publications	Citing (primary) publications that cite the same publications
Methodological mechanism	Publications are connected based on joint appearances on reference lists	Publications are connected based on the number of shared references
Time frame	Past	Present and future
Time-sensitive	Yes	No
Advantage	Enables a historic overview of the research field under study and identifies the most important publications	Detects the current state of research and enables the identification of future trends
Limitation	Because citations are needed to map publications, newer publications can be neglected	Harder to identify whether the mapped publications are important for the studied field

Co-citation analysis focuses on how primary publications cite pairs of secondary publications together. More specifically, it uses co-citation counts, which are defined as the number of times two publications are being cited together (Small, 1973), as a measure indicating semantic similarity (Vogel et al., 2021). The fundamental idea behind co-citation analysis is that the more two publications are cited together, the more content similarities they share. Publication co-citation identifies frequently cited publications even if they do not necessarily stem from the research field of interest—they might still be extremely important for its development. This property allowed us to identify highly-cited relevant publications not included in our database due to being published in books or journals that were not yet indexed at the time of publication. One notable limitation of co-citation analysis is that citations need time to accumulate because the publication process tends to be long and time-consuming. This means visualizations based on co-citations do not reflect the current state of the research field under study, but more the research field as it was some time ago.

Bibliographic coupling (Kessler, 1963) is a technique older than co-citation analysis (Small, 1973), although it is not used as often in bibliometric studies (Zhao & Strotmann, 2008). The focus is on primary publications rather than secondary ones since bibliographic coupling uses the number of references shared by two publications as a measure of similarity. This basically means that the more two bibliographies of two primary publications overlap, the greater their similarity. The technique addresses several drawbacks of co-citation analysis. Above all, since bibliographic coupling does not require citations to gather, it is situated in the present and allows for easier detection of research trends and priorities. It is also more suitable for the analysis of a smaller subfield, such as ours (Dominko & Verbič, 2019). Since citation habits change over time, it is reasonable to apply bibliographic coupling to a limited time frame, which is beneficial in our analysis since we are analyzing quite a young research field in which the majority of publications have been published in a limited time frame (Glänzel & Thijs, 2012). One notable limitation of bibliographic coupling compared to co-citation analysis is that it is harder to identify whether a mapped publication is important for the studied field; still, this issue can be resolved by simultaneously analyzing the citation count for each primary publication.

We used the VOSviewer software package to form and visualize the bibliometric networks, allowing us to conduct both a co-citation analysis and bibliographic coupling. The VOSviewer structures a bibliometric map in three steps. First, it uses a co-occurrence matrix to obtain a similarity matrix by correcting the matrix for differences in the number of occurrences or co-occurrences. Second, it constructs a map by locating items close to each other by minimizing the weighted sum of the squared Euclidean distances between all pairs of items. Finally, in the third step, it uses translation, rotation, and reflection to obtain consistent results. One item is assigned per cluster, while colors are used to distinguish different clusters. A detailed technical explanation and discussion of the method can be found in Van Eck and Waltman (2010, 2014b).

We complemented the VOSviewer analysis with the CitNetExplorer software package. CitNetExplorer visualizes both primary and secondary sources on a map where closeness between publications is highlighted on the horizontal axis and the year of publication on the vertical axis. As such, it helped us analyze the development of the research field and identify its antecedents. A detailed technical explanation of the software is given in Van Eck and Waltman (2014a).

### Evolutionary overview of the research field

**Fig. 1:**
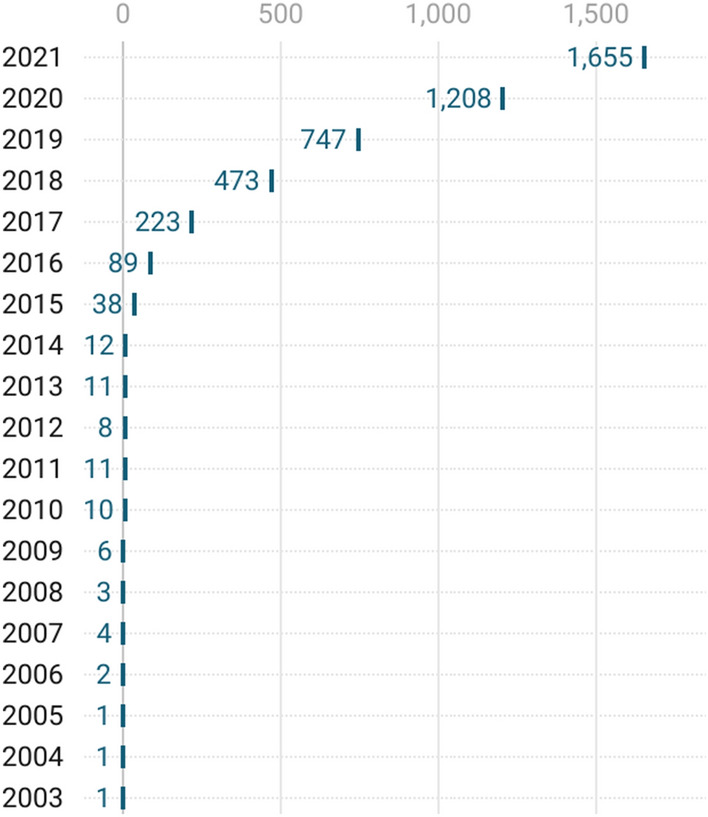
Number of articles on CE in the fields of business and economics by year

A descriptive overview of the obtained database reveals that the oldest article stems from 2003, indicating that research on CE in the fields of business and economics is still relatively young. Moreover, it was not until 2015 that the field started to grow exponentially. Between 2015 and 2021, the number of published articles rose by 4255%, from 38 published articles in 2015 to 1655 published articles in 2021 (Fig. 1). A look at the number of published articles in January 2022 shows this trend is not slowing down.

**Fig. 2:**
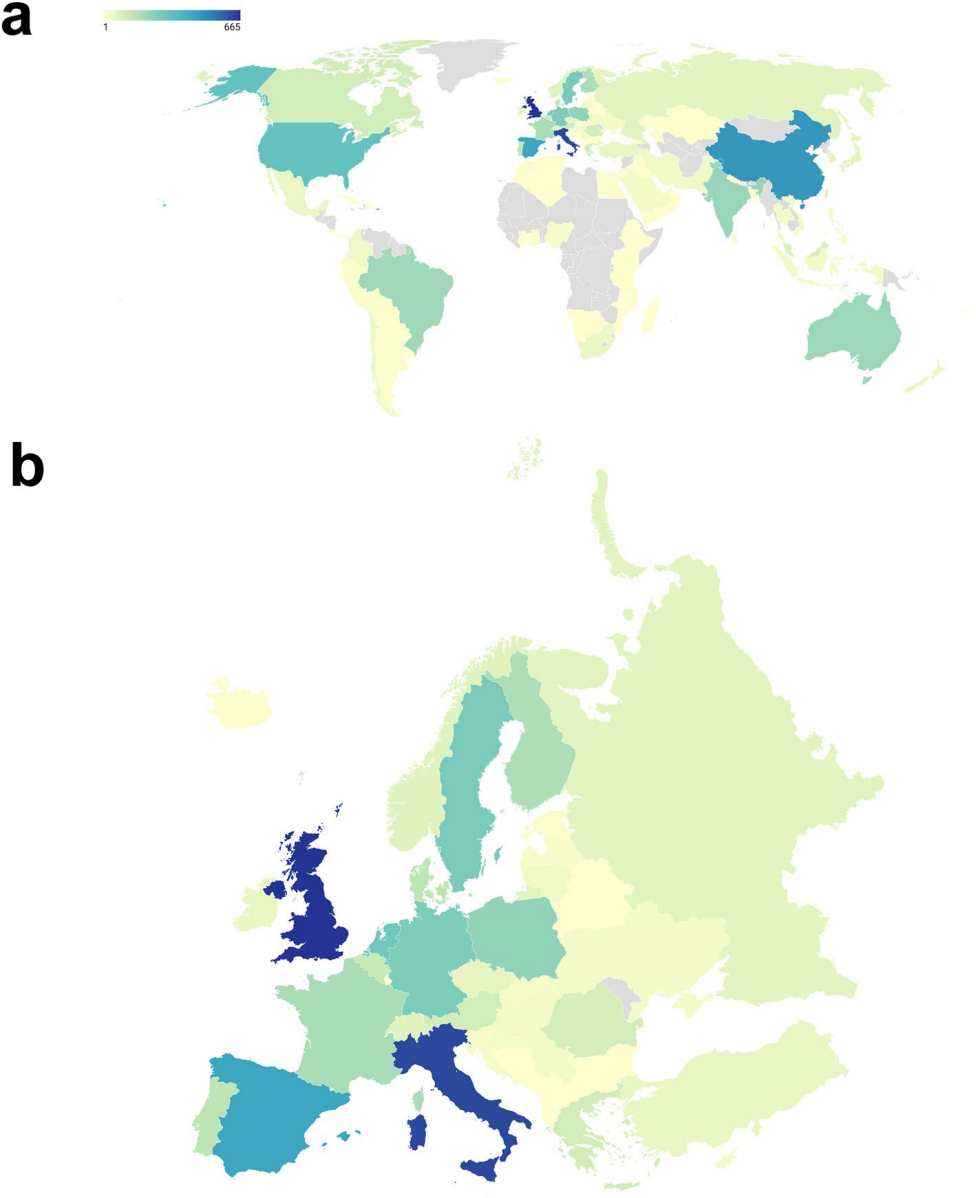
Countries publishing CE research in the fields of business and economics

During this period, the research field has also become substantially globalized. Figure 2 shows countries publishing CE research in the fields of business and economics. The diverging color scheme allows us to assess the global outreach of the research field and identify the most productive countries. The map reveals that new articles are emerging right across the globe. In general, Europe is the clear leader in the research field. The country with the most published articles in the United Kingdom (655 articles), followed by Italy (628 articles), China (474 articles), and Spain (436 articles).

**Fig. 3:**
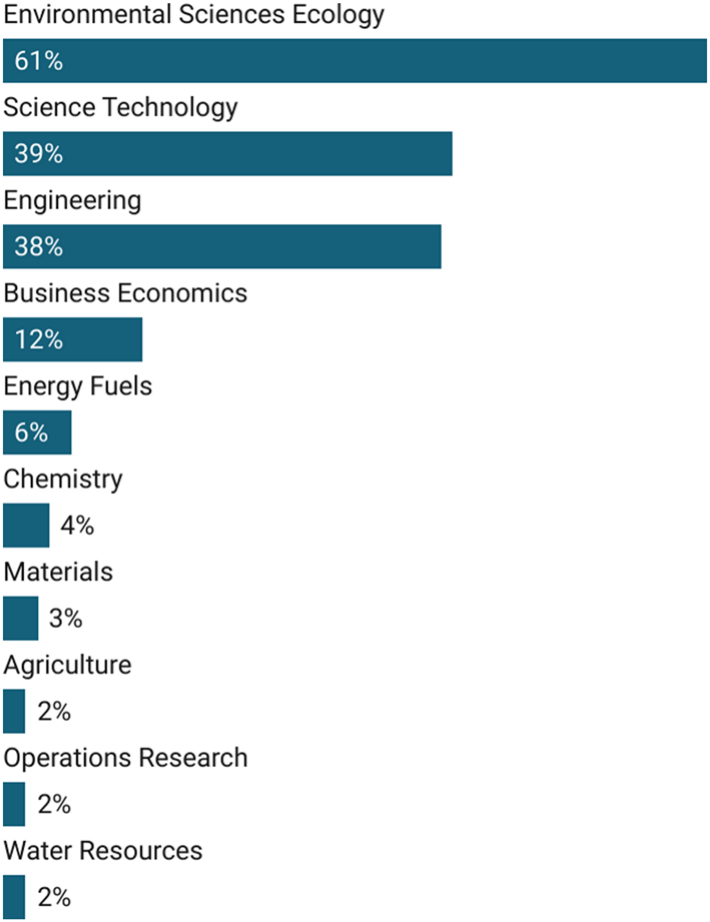
Research areas of CE research in the fields of business and economics

CE research in business and economics can be found in several research areas. Figure 3 shows the most frequent Web of Science research areas^2^ in which articles on CE in the fields of business and economics appear. Most articles are seen in the research area “environmental sciences ecology”, followed by the research areas “science-technology” and “engineering”. Interestingly, less than 15% of the published articles in our database is classified in the “business economics” research area, which highlights the interdisciplinary and transdisciplinary nature of the research field under study.

**Fig. 4:**
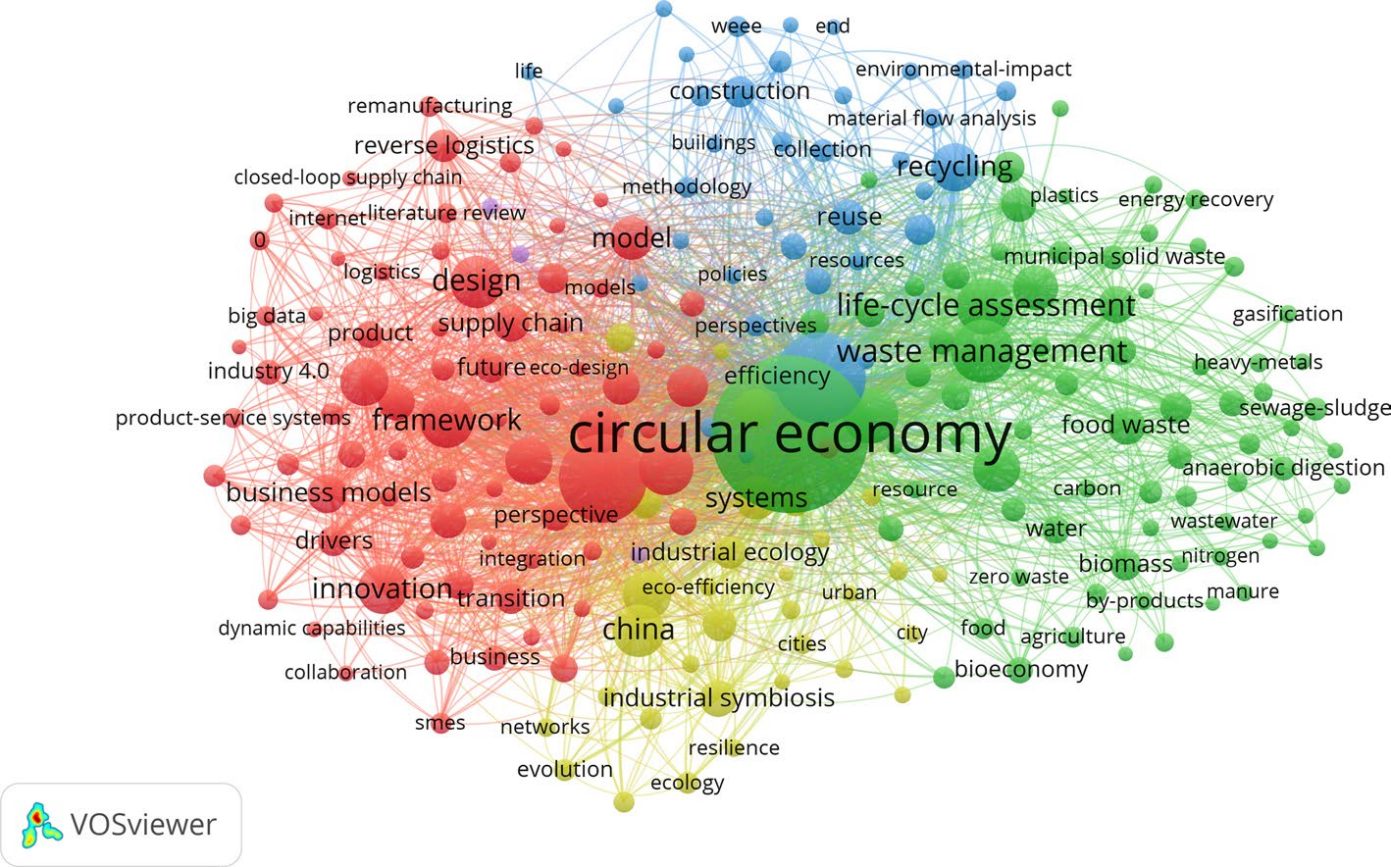
The most important keywords in CE research in the fields of business and economics

Analysis of the most important keywords, which provides a snapshot of the studied field, further confirms the observation that CE research in business and economics appears in several multidisciplinary and interdisciplinary contexts. We provide a visualization of the most important keywords in Fig. 4, where the size of a circle indicates the number of occurrences of a given keyword. The most important topics are concentrated in four clusters. The red cluster is the biggest and contains 94 keywords. A closer look at these keywords reveals that they refer to business models, innovation, integration, design, and framework. In general, these keywords revolve around the problem of translating theoretical CE ideas and innovations into practice. The second-biggest cluster is the green cluster, where keywords concern waste management, product life-cycle assessment, and emissions, as well as energy and resource recovery. These keywords suggest focus on the CE mostly on a technological level. The blue cluster may be seen as a bridge between the red and green clusters by containing keywords such as policies and methodology on the one hand, and recycling and material flow analysis on the other hand. Finally, the yellow cluster contains keywords, which chiefly concern CE topics studied in China, such as industrial symbiosis and industrial ecology.

**Fig. 5:**
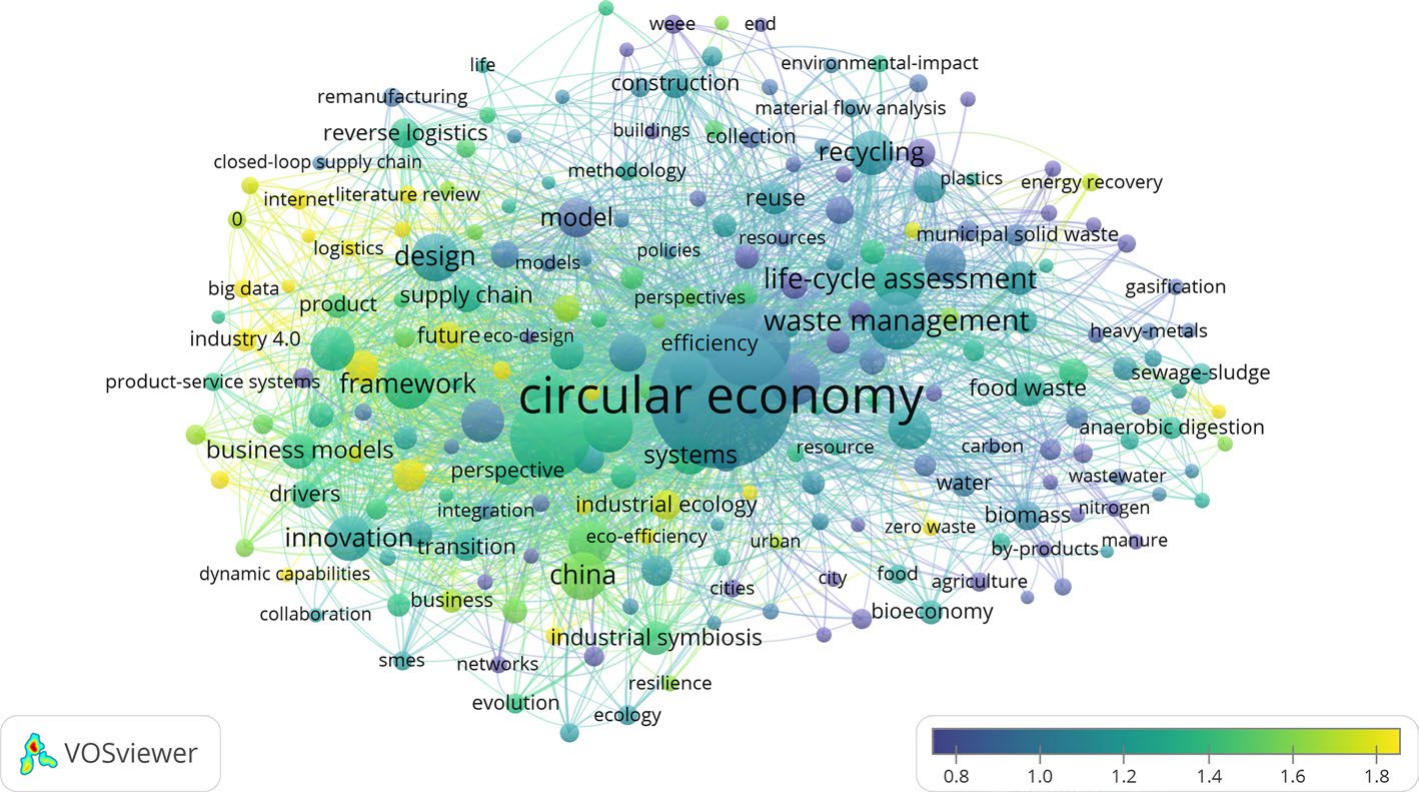
Overlay visualization of the most important keywords in CE research in the fields of business and economics

The number of occurrences is of course not the only property of the keywords presented. To understand which topics are being cited more often, we provide an overlay visualization in Fig. 5. In this visualization, keywords are colored according to their average normalized citation score, where the average citation score is divided by the mean score of all items. A comparison of the four clusters presented shows that, on average, the keywords in the red cluster have a significantly higher average normalized citation score than those in the green, blue and yellow clusters. Moreover, a closer look at the keywords with the highest scores reveals they refer to business models, the internet, and digitalization, which may be characterized as future-oriented. These findings allow us to conclude that CE research in the business and economics fields has shifted its focus from technology-related issues and is now more concerned with models and strategies bringing the CE to life.

### Past

**Fig. 6:**
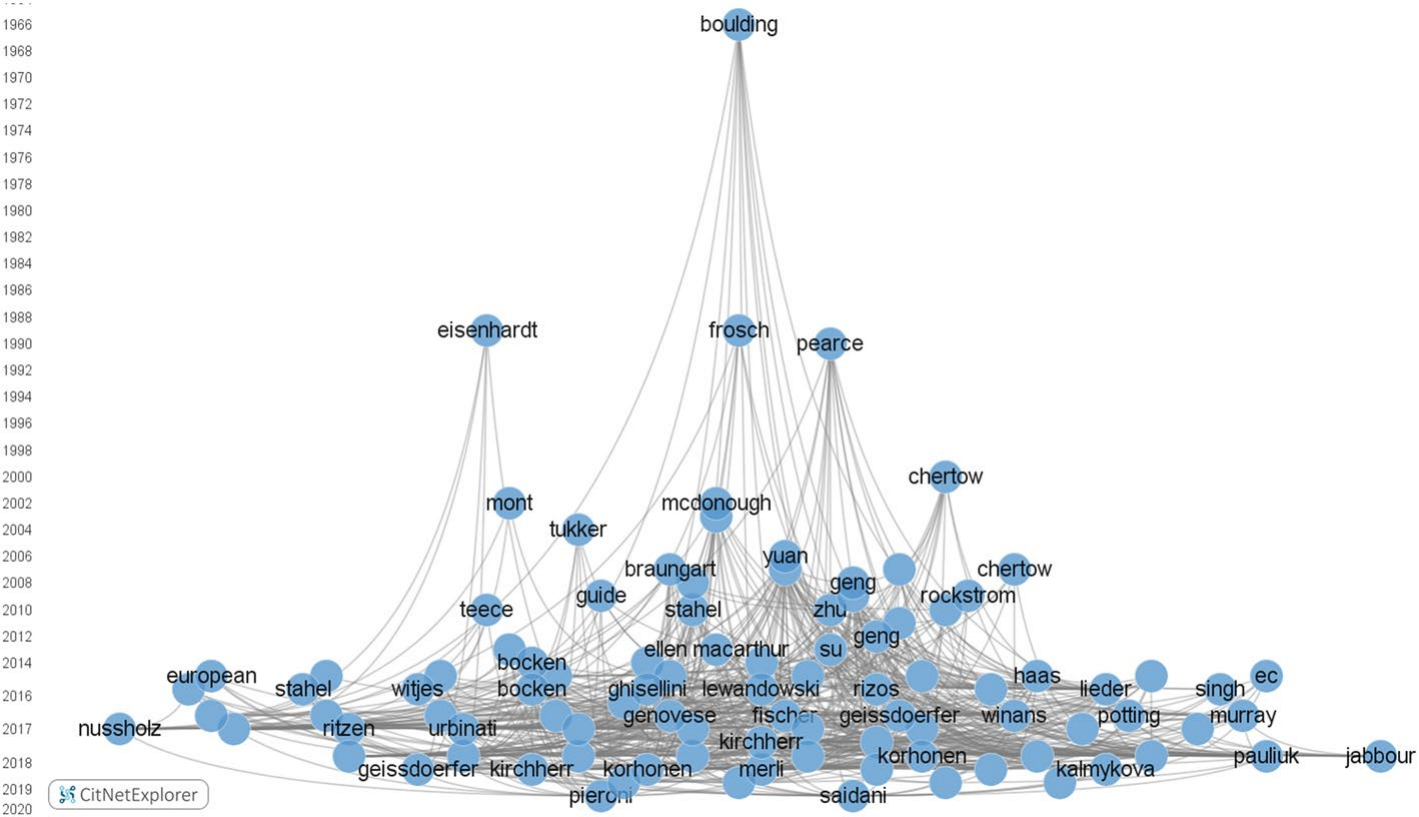
Citation network of the evolution of CE research in the fields of business and economics

To capture the evolution of CE research in business and economics, and also identify its antecedents, we used the software tool CitNetExplorer and drew a citation network based on all the primary and secondary publications in our dataset. Presented in Fig. 6, the results show that an important heavily cited predecessor can be found as early as 1966. In that year, Kenneth E. Boulding (1966) published his groundbreaking work “The Economics of the Coming Spaceship Earth”, in which he pointed out the need to adapt our economic system to the ecological system with its limited resources. His work influenced Pearce and Turner (1990), who introduced a modern understanding of CE systems by merging different features of a variety of concepts, like the laws of ecology (Commoner, 1971), industrial ecology (Graedel, 1994), regenerative design (Lyle, 1994), biomimicry (Benyus, 1997), cradle to cradle (McDonough & Braungart, 2002), looped and performance economy (Stahel, 2010), the blue economy (Pauli, 2010), and life cycle management and engineering (Niero et al., 2017). Another important forerunner of modern CE studies is the article by Rober A. Frosch and Nicholas E. Gallopoulos published in the Scientific American in 1989. In the article, the authors discuss how wastes from one industry can serve as the raw materials for another, thus providing an industrial ecosystem characterized by dematerialization and closed-system manufacturing. Finally, Fig. 6 reveals that predecessors that are not strictly theoretical in nature can nevertheless lead to important methodological findings. Such is the case with Kathleen M. Eisenhardt’s 1989 article, in which she describes the process of inducing theory using case studies, which had a significant impact on the methodology used in several CE studies in the business and economics fields.

### Present

**Fig. 7:**
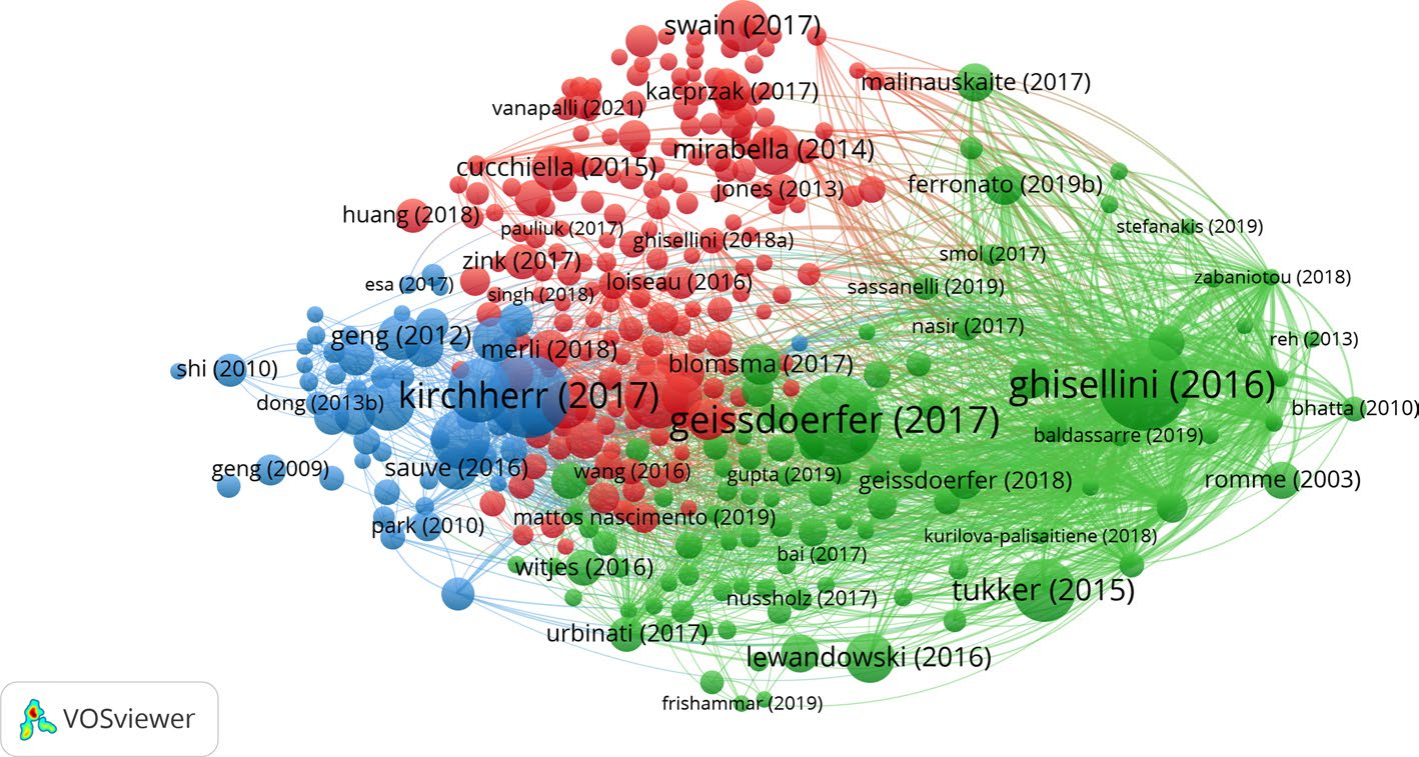
Citation network of articles on the CE in the fields of business and economics based on bibliographic coupling

Obtained with bibliographic coupling, the citation network of contemporary CE research in business and economics in Fig. 7 reveals three major clusters of articles. These indicate three key directions in which research is heading and allow us to draw several parallels with the previous analysis of the most important keywords. The blue cluster mainly features articles that perceive and study the CE on the macro level. One of the key themes in this cluster is industrial symbiosis. Most of the research on industrial symbiosis stems from China due to its consensus on the CE concept, which underlines the advantages of using residual waste materials like water, energy, or even information. Industrial symbiosis, where collective benefits arise from both the environment and the economy, is the most common example (Su et al., 2013). Other topics found in the blue cluster are also linked to China and concern the institutional change needed for the successful implementation of CE practices (Ranta et al., 2018; Wang et al., 2017), as well as transdisciplinary and interdisciplinary explorations of the CE concept (Murray et al., 2017; Sauvé et al., 2016). Unlike the blue cluster, the green cluster mainly contains articles exploring CE business models and consequently CE on the mezzo and micro levels. The articles explore several aspects of CE business models, such as their implementation in theory and practice, their potential and their practical implications. Further, several articles in the cluster have already moved from the general to the specific by addressing CE business models in certain industries like construction or apparel (Adams et al., 2017; Stål & Corvellec, 2018), which is very important for the broader implementation of CE business models, and for CE business model innovation. A closer observation of the red cluster shows it is strongly intertwined with the green cluster and that it deals with CE on the mezzo and micro levels. This is reflected in a wider array of topics compared to the other two clusters. Like in the green cluster, several articles deal with CE business models; however, in the red cluster, they are more closely linked to the concepts such as big data, and industry 4.0. (Bag et al., 2021; de Sousa Jabbour et al., 2018). Moreover, the cluster is concerned with the CE as a concept and highlights its limitations and drawbacks (Korhonen et al., 2018; Reike et al., 2018). Finally, there is overlap among all three clusters regarding supply chain management, as well as reviews, which are among the most cited articles (Ghisellini et al., 2016; Zhu et al., 2010). A detailed list of the most heavily cited articles in the area of the CE in business and economics may be found in the appendix (Table 3).

Case studies play a noticeable role in each of the three main clusters of articles because they permit a well-rounded overview of certain CE practices. Here, the extensive use of case studies in China is quite evident. Such case studies may be placed in two groups, where the former analyzes particular Eco-industrial Parks (EIP) in the country, namely a core element for helping to simultaneously support industrial growth while reducing the impact on the environment (Geng et al., 2010; Shi et al., 2010). In contrast, the latter group delves into certain sectors or companies that have successfully woven the CE philosophy into their material and energy flows as part of moving towards development which is sustainable (Yang & Feng, 2008). Although case study research concerned with the CE in the economics and business areas sees China in the lead, the insights found in case studies are being absorbed by academics all around the world. These contributions refer to a wide spectrum of cases, like CE practices used in the building sector (Leising et al., 2018), the search for rare earth elements (Kulczycka et al., 2016), CE business models (Bocken et al., 2018), sustainable tourism (Scheepens et al., 2016), also including how household waste is sorted (Miliute-Plepiene & Plepys, 2015).

**Fig. 8:**
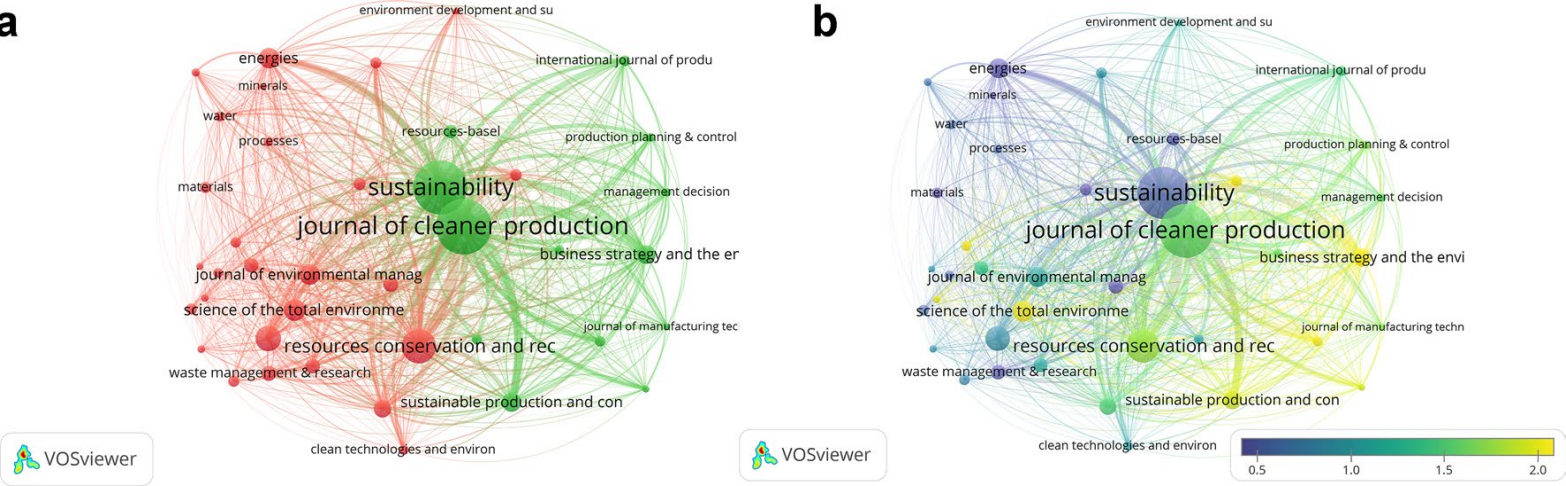
Citation network and overlay visualization of journals publishing articles on CE in the fields of business and economic-based on bibliographic coupling

Bibliographic coupling can be extended to authors, journals, organizations, or even countries, and can thus offer additional insights into CE research in business and economics. Especially interesting is a closer look at the journals publishing research on the CE in the business and economics fields, which, first of all, shows that the three most productive journals are the *Journal of Cleaner Production* (669), *Sustainability* (622) and *Resources, Conservation & Recycling* (251).^3^ Figure 8 shows the citation network of journals based on bibliographic coupling and reveals two clusters of journals publishing CE research in business and economics. While these two clusters share several characteristics and all three leading journals convey the vast majority of topics explored in the field, there is an important distinction between them. On one hand, the red cluster is on average more oriented to waste and environmental management, as well as materials. On the other hand, the green cluster is on average more business-model-oriented. The distinction is also visible in the overlay visualization given in Fig. 8, which sheds extra light on the topic. It reveals that journals in the green cluster have a higher average normalized citation score than those in the red cluster, providing further evidence that ever more attention is being given to the transition of CE research from theory to practice.

### Future

In order to identify gaps and future trends in CE research in business and economics, we performed a qualitative review of our database and identified research articles with the highest usage count in the last 180 days. Usage count, which measures the level of interest in a research article logged by Web of Science, reflects the number of times an article has been accessed or saved in the last 180 days (in our case, the observed period spans from August 4, 2021, to January 31, 2022). It enables us to address the drawback of using citations as the primary measure of an article’s impact since citations require time to accumulate due to the prolonged publishing process and thus often do not accurately reflect the present situation.

A list of articles with the highest usage counts (excluding review articles) is given in Table 2 along with their citation counts. The list demonstrates that the most cited articles are not necessarily also those that have been used the most recently. There are many articles on the list with hardly any or no citations, yet they have received a lot of recent attention from academia and real-world practice. These articles explore three main hotspots: sustainable supply chains, waste management, and business model innovation.

**Table 2:** Articles on the CE in the fields of business and economics with the highest 180-day usage count

Authors	Article	Journal	Year	Cit.	Usage (180)
Ranta, V.; Aarikka-Stenroos, L.; Vaisanen, J.M	Digital technologies catalysing business model innovation for circular economy–Multiple case study	Resources, Conservation & Recycling	2021	23	145
Nascimento, D.L.M.; Alencastro, V.; Quelhas, O.L.G.; Caiado, R.G.G.; Garza-Reyes, J.A.; Lona, L.R.; Tortorella, G	Exploring Industry 4.0 technologies to enable circular economy practices in a manufacturing context: A business model proposal	Journal of Manufacturing Technology Management	2019	174	87
Genovese, A.; Acquaye, A.A.; Figueroa, A.; Koh, S.C.L	Sustainable supply chain management and the transition towards a circular economy: Evidence and some applications	Omega	2017	427	86
Bag, S.; Gupta, S.; Kumar, S	Industry 4.0 adoption and 10R advance manufacturing capabilities for sustainable development	International Journal of Production Economics	2021	59	83
Bag, S.; Pretorius, J.H.C.; Gupta, S.; Dwivedi, Y.K	Role of institutional pressures and resources in the adoption of big data analytics powered artificial intelligence, sustainable manufacturing practices and circular economy capabilities	Technological Forecasting and Social Change	2021	56	81
Geissdoerfer, M.; Morioka, S.N.; De Carvalho, M.M.; Evans, S	Business models and supply chains for the circular economy	Journal of Cleaner Production	2018	244	76
Kirchherr, J.; Piscicelli, L.; Bour, R.; Kostense-Smit, E.; Muller, J.; Huibrechtse-Truijens, A.; Hekkert, M	Barriers to the circular economy: Evidence from the European Union (EU)	Ecological Economics	2018	316	75
Kristoffersen, E.; Mikalef, P.; Blomsma, F.; Li, J.Y	The effects of business analytics capability on circular economy implementation, resource orchestration capability, and firm performance	International Journal of Production Economics	2021	7	73
Zink, T.; Geyer, R	Circular economy rebound	Journal of Industrial Ecology	2017	240	71
Qureshi, M.S.; Oasmaa, A.; Pihkola, H.; Deviatkin, I.; Tenhunen, A.; Mannila, J.; Minkkinen, H.; Pohjakallio, M.; Laine-Ylijoki, J	Pyrolysis of plastic waste: Opportunities and challenges	Journal of Analytical and Applied Pyrolysis	2021	74	69

#### Peculiarities of sustainable supply chains

While there is a general consensus in academia that companies are crucial for the transition to CE (Kalmykova et al., 2018), systemic changes are needed if we are to effectively include all actors at different points of the supply chain that add value to a product or a service (Elia et al., 2020). However, studies on processes which constitute supply chain management in CE business models are scarce (Vegter et al., 2020). This is a big limiting factor since the diffusion of the CE depends on a coherent understanding of the new approaches, methods, and tools that support an integrated approach along an individual company’s entire supply chain and determine the performance of its accompanying strategies. Specifically, one fundamental difference between a linear supply chain and a circular supply chain is that one needs to cover both the forward and the reverse flows of the supply chain. In addition to technical and cultural barriers, operating within a linear business model is one of the most pressing barriers to CE implementation (Kirchherr et al., 2018). Companies will have to adapt activities to the new system (e.g. reverse logistics) and their interplay. Big data analytics-powered artificial intelligence has already proven its effectiveness in managing supply chains (Bag et al., 2021; Chien et al., 2020). Therefore, future research should conceptualise circular supply chain management and design suitable performance measures. The task is challenging since the processes must be economically viable while grounded in sustainability principles (Zink & Geyer, 2017).

#### Waste management: a step back to assessing end-of-life values

Opportunities for cost-saving by reducing waste are offered at every stage of a product’s life cycle, from production to the recovery of products that are not used or resold (Morseletto, 2020). Waste reduction is a fundamental part of the CE, which is very popular among scholars who cover general topics, such as measuring economic performance regarding waste flows (Wang et al., 2020), waste management challenges imposed by COVID-19 (Neumeyer et al., 2020), and opportunities for developing an efficient country-wide waste management system (Fatimah et al., 2020; Qureshi et al., 2020), as well as among those who cover narrow topics concerning the types of waste (e.g. marine debris (Agamuthu et al., 2019), commercial plastic waste (Khan et al., 2020), food waste (Oldfield et al., 2016), wood waste (Berger et al., 2020)), municipal solid waste collection systems (Calabro & Satira, 2020; Ferronato et al., 2019), and bio-waste management options on the city and regional level (Zeller et al., 2020). Although the development of this field has been rapid, practitioners lack access to information about the end-of-life values of products required for the efficient planning of the materials’ reuse and recycling to build on targets to steer the transition from a linear to a circular state. While some practical data might already be available to policymakers and managers (e.g. Akanbi et al., 2020; Bahn-Walkowiak & Steger, 2015; Morseletto, 2020), future research should take a step back and consider studying targets for specific processes, products, and industries, as well as designing machine learning models to address what is required for estimating and predicting different products’ end-of-life values before moving on to new methods and technological solutions.

#### Circular business model innovation: testing and improving existing models

A circular business model is generally any business model that captures the notion of the circularity of resources in a closed loop (Chen et al., 2020). Although research on circular business models has been expanding, more effort is needed to advance our knowledge of how companies operate in the CE (Urbinati et al., 2020). Specifically, if we want to better understand how companies create, capture, and deliver value in the CE, we must collect evidence from practice (Pieroni et al., 2020). Therefore, today’s prime goal should be to test and improve the already developed models in order to strengthen their empirical suitability in national and international contexts. In doing so, we should not ignore external contingencies (Morris et al., 2005; Primc & Čater, 2015), those associated with institutional pressure and regulation, and their role in shaping CE business models. Other important venues for future research include the development of business management tools used to analyze the environmental impact of circular business models and digital technologies from Industry 4.0 that can enable adhering to CE. Currently, popular products of the linear economy, like the business model canvas, raise concerns regarding their applicability to the CE due to their lack of a systematic perspective and focus on a single business (Chen et al., 2020). Accordingly, the existing analytical tools need to be applied only at specific applicable stages (e.g. solution availability, solution development, solution evaluation) and/or when adequately adapted to fit the current circular paradigm. On the other hand, the lack of empirical studies in the existing articles reduces the potential for drawing broader implications on how digital technologies enable and trigger CE business model innovation. Digital technologies are crucial in the fourth industrial revolution, called Industry 4.0 (Lieder & Rashid, 2016).

## Step 2: Qualitative literature review of action-oriented research in CE research

Action-oriented research (also known as action research) is qualitative social research conducted by a group that includes researchers, representatives of companies (e.g. managers, designers, engineers), authorities, and communities that wish to improve their situation. Action research solves problems, tests knowledge, and creates new knowledge at the same time. It aims to obtain information and tests it by connecting theory and practice. All stakeholders should contribute their skills to increase the sustainable and circular practices of both the economy and the community (Kumble, 2019). In other words, researchers and stakeholders need to collaborate on the production of knowledge. Action research is an alternative to the classical approach where a researcher is an independent observer who collects data, analyzes it, and seeks answers to research questions posed by the science itself. In contrast, in action research, a group of stakeholders identifies the research question through a participatory process and together creates a solution via mutual interaction. In the case of research in the CE field, a co-design or co-innovation process of products/services is often carried out. This process is intended to acquire scientific knowledge for the transition to the CE and the actual realization of this transition (Christensen, 2021). Namely, it is crucial to constantly test theoretical tools and communicate good practices to researchers and companies engaged in CE research. Only in this way will it be possible to move to a higher level of circularity and sustainability (Saidani et al., 2020).

### Action-oriented research on the circular economy

To investigate how the research on the CE can better support companies and be successfully implemented in practice, we expanded the database of studies to the Scopus Collection, concentrating on action-oriented research. We ran search queries on our primary topic with the search term “circular economy” (the number of search results did not change when synonyms were used for the CE, i.e. “closed-loop economy” and “zero waste economy”) and limited our search to articles that focus on the CE and action-oriented research by including the additional search term “action research”. Its synonyms, i.e. “action-oriented research”, “participatory action research”, and action-led research” once again did not have any impact on the search results. This process returned 28 articles, of which 6 articles were excluded due to different research contexts. The searched studies demonstrate that action-oriented research at the intersection of the CE and business and economics creates value for academia, industry, society, and the environment. Still, a small number of studies have emerged, but only recently. This means the action research approach has only recently begun to take root in the analyzed field. Table 5 in the appendix clusters the combined insights of action-oriented research according to the three hotspots identified in Step 1: sustainable supply chains, waste management with an assessment of end-of-life values, and innovative CE business models.

Action projects on sustainable supply chains generally focus on closing the loop in the food value chain, circular procurement, citizens’ projects with activities and places for urban agriculture, recycling and reuse, community energy production, etc. (Cramer, 2020; Nogueira et al., 2020; Petrescu et al., 2016). One example of good practice is the French R-Urban model. Researchers, architects, and public and civilian actors have worked together to create a network of civic hubs to support practices, tools, spaces, and actors to increase climate resilience. Citizens were not only participants in these projects but also agents of innovation and change. R-urban generated social, ecological, and economic impacts such as the reduction of waste by 500 *t* per year, the reduction of CO_2_ emissions by 300 *t*, 70% of locally produced electricity, turnover of 100,000€/year, temporary green jobs, and 8 international exhibitions (Petrescu et al., 2016).

Existing theories on the CE have already been modified according to findings obtained by applications to resolve practical waste management problems. An action researcher and representatives of companies or communities have been involved in the research. Research has been conducted in the fields of composting, bio-related technologies, value creation, and end-of-life management of heavy vehicles, textile recycling, waste management in a chemical plant, demolition practices of buildings, etc. (Kumble, 2019; Lag-Brotons et al., 2020; Saidani et al., 2020; Christensen, 2021; Becerra et al., 2020). A case study of a rural community in Mexico shows that a self-sustainable community can be achieved by converting residue, waste, and by-products into returning cycles. The research team found that 137 L of cheese whey were being discarded at a cheese factory and that the best option was to use the cheese whey to produce isotonic beverages. Further, the viability of a hydro-distillation process to extract essential oils from wasted oregano stems at an oregano plantation was examined. The plantation owner is already considering the market opportunities of selling these essential oils. Moreover, the team evaluated the feasibility of scaling up the red worm farm and increasing the demand for compost from community/urban gardens. The team also proposed transforming the wood residues from the lumber mill into biochar through pyrolysis. This biochar would then be mixed with compost and used to improve soil properties (Aguiñaga et al., 2018).

Circular business models comprise the operation and organization of the company that creates and delivers value (product/service) to the consumer while bringing social and environmental benefits by focusing on slowing, closing, and narrowing resource loops. Businesses must experiment with their circular business models to retain a competitive advantage in a rapidly changing economic environment (Bocken et al., 2017). Research experimenting with innovative circular business models was carried out in various sectors, such as capital goods, furniture, electronic equipment, textile and clothing, medical devices, consumer durables, agriculture, a product-sharing platform, drinking water, and tailored water services, etc. (Bocken et al., 2018; Pieroni et al., 2019). For example, Real et al. (2020) explored the local business model for a regional transition to circular textiles in France. In the case of a co-working community of sewers, the project enabled four gains: a collective co-working space, shared service provision, the offering of courses and additional commercial activities. Further, the innovative circular business model of the industrial upcycling center enabled the use of textile scraps for cushion paddles in eco-designed furniture. The project supported the employment of 19 persons and the collection, recycling, upcycling, or reusing of 145 tonnes of waste.

## Discussion

After 7 years of CE studies seeing exponential growth, one might ask: How is it possible that scholars have missed anything? Our findings suggest that while the theoretical progress is remarkable and the analytical approaches sophisticated, companies must discuss practices and guidelines with their key stakeholders if they are to successfully transform their operations. Therefore, we believe that scholars must re-focus on action-oriented research to ensure that the CE is as impactful as suggested in the literature. As CE scholars, we have to accept the role of active agents that help companies redesign their operations.

Our study contributes to the literature in several ways. First, by providing a bibliometric map of the CE research in business and economics we were able to objectively trace the evolution of the research field, which plays a crucial role in implementing CE practices in companies. Compared to previous studies, which do not focus exclusively on the CE in business and economics, we have combined several techniques, such as co-citation analysis and bibliographic coupling, as well as keyword co-occurrence and usage count. This allowed us to objectively analyze the past, present, and future of the field in question, adding specific insights to previous knowledge on the CE in the domains of business and economics. Our results show that while the research field is relatively young and has been exponentially expanding over the last 5 years, it has clear antecedents that can be dated back to as far as 1966. These have provided relevant pathways, which continue to be used by contemporary researchers. In general, articles study the CE on the macro level on one hand, and on the mezzo and micro levels on the other. While the field is concerned with several topics that can be grouped into three clusters, the trend indicates that ever more scholars are addressing issues associated with business models, indicating a desire to transition from theory to practice.

Second, we detected the existing research gaps and outlined future directions to guide managers willing to embrace the CE. Although progress in the theoretical field is impressive, companies are particularly interested in the practical potential held by the concept. Therefore, it seems that the current missing practical aspect hinders any faster transition to the CE. Based on our qualitative review of the database and the highest usage count in the last 180 days, we identified 3 areas that deserve priority in future studies. These include elaborating on the processes that constitute supply chain management, developing waste management targets and data-driven models for predicting products’ end-of-life and putting the existing circular business models into practice. Our findings also indicate that the topics of the future are gradually shifting away from the micro or single-company perspective toward a systemic perspective. While in the study conducted by Kirchherr et al. (2018) only around 40% of the definitions envisage the CE from a systemic perspective, this will arguably change in the near future. Several studies (e.g. Bauwens et al., 2020) have already clarified that the transition to the CE must happen on the systemic level, where the operation of companies is strongly intertwined and interdependent. Finally, while the impact of the COVID-19 pandemic on research topics is not yet apparent in the current article, it is expected that future studies will intensively examine CE business operations in uncertain environments, emerging sectors, and the transformation of those existing sectors worst affected by the pandemic, such as tourism that is restorative.

Third, in terms of the content, the existing action research is being conducted in the areas we identified as hotspots. We observe that CE scholars mostly focus on one of two aspects: the techno-economic or the organizational aspect. From a techno-economic perspective, researchers work with stakeholders to develop: (1) ways to create optimal circular material and energy flows considering the associated costs; (2) co-design processes for products/services; and (3) co-innovation processes for products and services. These types of research are generally conducted as industrial pilot studies, as well as in collaboration with university spin-offs. Diversely, as part of the organizational aspect, research deals with an internal or external organization, self-sustainable community network, and participatory governance. These aspects are often intertwined and implemented in innovative circular business models.

For companies, the CE transformation is not easy, but many low-hanging fruits can be harvested to encourage this transition. From the point of view of academia, the real world can be approached with secondary data to receive as much information about what pays the most while complying with the principles of the CE. Ideally, CE scholars could gather knowledge about the most effective solutions to a particular issue by designing several measurable pilot programs to determine their effectiveness in different settings. Still, companies are the ones that best know their challenges and possibilities regarding improvement. Their willingness to cooperate closely with academia and other relevant stakeholders is necessary to produce high-quality and relevant information. However, they need to be challenged first in order to change their current mindsets and switch over to longer-term visions. One way to do this is to anticipate future developments with quantitative modeling, helping companies understand the consequences of a new paradigm (Bauwens et al., 2020). Insights about alternative CE futures provide important information for companies in the planning stage of the transformation and policymakers in shaping effective policies.

While collaboration between companies and researchers is crucial for the successful implementation of CE practices, we must not underestimate the importance of other stakeholders, especially policymakers. The role of policymakers is first to ensure key policy directions. These include: the adoption of stable long-term policies that reduce uncertainty and allow for business model innovation; the reduction of single-use and hazardous materials in products; as well as the development of CE infrastructure in support of decarbonization. Further, regulatory actions must be developed, such as taxation, reporting, extended producer and consumer responsibility, product bans or standards, etc. To deliver an effective framework, it is crucial that governments collaborate with several partners across all of society to ensure the successful integration of policies and regulations with scientific research. Particularly important is the collaboration between governments and academia. According to Velenturf et al. (2018), the role of academia is first to identify policies linked to a specific CE project, second to perform a situational analysis to understand if a new approach or technology could be realized within the policy and regulatory context, and third to explicitly connect solutions and recommendations to policies and regulations in a specific region. Finally, researchers should provide the ‘bigger picture’ and constantly communicate with governments to assure the timely recommendations needed in the decision-making process.

The idea of an ecological transformation is several decades old, yet we are only now seeing noticeable progress in greening the economy. It is about the whole of society maturing, and this is the fundamental difference between the existing transformation and less successful attempts in the past. Together with the experience of COVID-19, which is perhaps the most enlightening, disruptive, and instructive event demonstrating the importance of sustainability, we seem to be ready for the new paradigm.

## Limitations and conclusion

Academic articles have become a considerable source of knowledge on the CE with valuable theoretical contributions, yet what we are currently witnessing is only a slow transition to the CE in practice. On this basis, one goal of this article was to elucidate the reasons for this reluctant progression by identifying the mismatch between theoretical opportunities as anticipated in the literature and the substantive implementation undertaken by companies. Together with pinpointing the most important publications, authors, journals, and keywords, we have contributed to a better understanding of past and current advancements in the field and illuminated future pathways to expedite the micro-level transition to the CE according to the latest findings. Like with most articles, ours is not immune to certain limitations, which, despite taking all the necessary precautions, mostly concern the database used. First and foremost, because we drew publication and citation data for the bibliometric analysis exclusively from the Web of Science website, we were unable to systematically validate the selection bias that might have occurred, which means we ran the risk of omitting relevant articles from our database. Second, the construction of the database focused solely on CE research in the fields of business and economics. However, new terminology has recently started to appear that complements the term “circular economy” and could thus be integrated in future analyses. Finally, the bibliometric analysis could be extended to other databases, methodological approaches, and types of publication. Despite these limitations, we were able to provide an objective, comprehensive, and integrative overview of CE research in the fields of business and economics.

## Appendix

(See Tables 3, 4, 5).

**Table 3:** The most cited articles on CE in the fields of business and economics

Authors	Title	Journal	Year	Cit
Ghisellini, P.; Cialani, C.; Ulgiati, S	A review on circular economy: The expected transition to a balanced interplay of environmental and economic systems	Journal of cleaner production	2016	1538
Geissdoerfer, M.; Savaget, P.; Bocken, N.M.P.; Hultink, E.J	The circular economy—A new sustainability paradigm?	Journal of cleaner production	2017	1494
Kirchherr, J.; Reike, D.; Hekkert, M	Conceptualizing the circular economy: An analysis of 114 definitions	Resources, conservation and recycling	2017	1284
Korhonen, J.; Honkasalo, A.; Seppala, J	Circular economy: The concept and its limitations	Ecological economics	2018	773
Lieder, M.; Rashid, A	Towards circular economy implementation: A comprehensive review in context of manufacturing industry	Journal of cleaner production	2016	756
Tukker, A	Product services for a resource-efficient and circular economy—A review	Journal of cleaner production	2015	740
Murray, A.; Skene, K.; Haynes, K	The circular economy: An interdisciplinary exploration of the concept and application in a global context	Journal of business ethics	2017	654
Su, B.; Heshmati, A.; Geng, Y.; Yu, X	A review of the circular economy in China: Moving from rhetoric to implementation	Journal of cleaner production	2013	508
Swain, B	Recovery and recycling of lithium: A review	Separation and purification technology	2017	500
Lewandowski, M	Designing the business models for circular economy-Towards the conceptual framework	Sustainability	2016	470
Mirabella, N.; Castellani, V.; Sala, S	Current options for the valorization of food manufacturing waste: A review	Journal of cleaner production	2014	470
Genovese, A.; Acquaye, A.A.; Figueroa, A.; Koh, S.C.L	Sustainable supply chain management and the transition towards a circular economy: Evidence and some applications	Omega	2017	427
Geng, Y.; Fu, J.; Sarkis, J.; Xue, B	Towards a national circular economy indicator system in China: An evaluation and critical analysis	Journal of cleaner production	2012	385
Kalmykova, Y.; Sadagopan, M.; Rosado, L	Circular economy—From review of theories and practices to development of implementation tools	Resources, conservation and recycling	2018	372
Cucchiella, F.; D'Adamo, I.; Koh, S.C.L.; Rosa, P	Recycling of WEEEs: An economic assessment of present and future e-waste streams	Renewable and sustainable energy reviews	2015	371
Sauve, S.; Bernard, S.; Sloan, P	Environmental sciences, sustainable development and circular economy: Alternative concepts for trans-disciplinary research	Environmental development	2016	336
Korhonen, J.; Nuur, C.; Feldmann, A.; Birkie, S.E	Circular economy as an essentially contested concept	Journal of cleaner production	2018	325
Merli, R.; Preziosi, M.; Acampora, A	How do scholars approach the circular economy? A systematic literature review	Journal of cleaner production	2018	317
Kirchherr, J.; Piscicelli, L.; Bour, R.; Kostense-Smit, E.; Muller, J.; Huibrechtse-Truijens, A.; Hekkert, M	Barriers to the circular economy: Evidence from the European Union (EU)	Ecological economics	2018	316
Blomsma, F.; Brennan, G	The emergence of circular economy: A new framing around prolonging resource productivity	Journal of industrial ecology	2017	316

**Table 4:** Top 20 journals with the most publications in CE research in the fields of business and economics

Journal	Articles	JCR IF 2020
Journal of cleaner production	669	9.297
Sustainability	622	3.251
Resources, conservation and recycling	251	10.204
Waste management	142	7.145
Science of the total environment	103	7.963
Energies	93	3.004
Journal of environmental management	91	6.789
Sustainable production and consumption	82	5.032
Business strategy and the environment	74	10.302
Journal of industrial ecology	66	6.946
Waste Management & Research	51	3.549
Applied sciences—basel	49	2.679
Renewable and sustainable energy reviews	48	14.982
Environmental science and pollution research	46	4.223
Resources—basel	41	-
Ecological economics	34	5.389
Detritus	29	-
International journal of environmental research and public health	28	3.390
Journal of material cycles and waste management	27	2.863

**Table 5:** Results of the literature review on circular economy and action-oriented research

Authors	Title	Journal	Year	Hotspots (details)	Participants in the research
Cramer J.M	Implementing the circular economy in the Amsterdam metropolitan area: The interplay between market actors mediated by transition brokers	Business Strategy and the Environment	2020	Sustainable supply chain (circular procurement)	Local government, industry, research institutes, university
Cramer J.M	Practice-based model for implementing circular economy: The case of the Amsterdam metropolitan area	Journal of Cleaner Production	2020	Sustainable supply chain (circular procurement)	Local government, industry, research institutes, university
Nogueira A.; Ashton W.; Teixeira C.; Lyon E.; Pereira J	Infrastructuring the circular economy	Energies	2020	Sustainable supply chains (food)	Research institute, plant, community (urban agriculture & food businesses)
Petrescu D.; Petcou C.; Baibarac C	Co-producing commons-based resilience: Lessons from R-Urban	Building Research and Information	2016	Sustainable supply chain (citizens projects)	Community
Christensen T.B	Towards a circular economy in cities: Exploring local modes of governance in the transition towards a circular economy in construction and textile recycling	Journal of Cleaner Production	2021	Waste management (demolition materials & textile wastes)	Municipalities, waste companies, research institutes
Saidani M.; Yannou B.; Leroy Y.; Cluzel F	Dismantling, remanufacturing and recovering heavy vehicles in a circular economy—Technico-economic and organizational lessons learnt from an industrial pilot study	Resources, Conservation and Recycling	2020	Waste management (end of life options—heavy vehicles)	International remanufacturing centre of heavy handling machines
Becerra L.; Carenzo S.; Juarez P	When circular economy meets inclusive development. Insights from urban recycling and rural water access in Argentina	Sustainability (Switzerland)	2020	Waste management (industrial waste); water supply – use & reuse of water)	Plant staff & waste picker organizations; community (households/farmers & public
officers & teachers from the local school					
Lag-Brotons A.J.; Velenturf A.P.M.; Crane R.; Head I.M.; Purnell P.; Semple K.T	Editorial: Resource recovery from waste	Frontiers in Environmental Science	2020	Waste management (bio-related technologies)	Industry, politicians, NGOs, communities, academia
Kumble P.A	Reflections on service learning for a circular economy project in a Guatemalan neighborhood, Central America	Sustainability (Switzerland)	2019	Waste management (compost)	Community, start-up, university
Wu H.; Wu R	The role of educational action research of recycling process to the green technologies, environment engineering, and circular economies	International Journal of Recent Technology and Engineering	2019	Waste management (3D printing design)	Consumer, 3-D printing manufacturers
Velenturf A.P.M.; Purnell P.; Tregent M.; Ferguson J.; Holmes A	Co-producing a vision and approach for the transition towards a circular economy: Perspectives from government partners	Sustainability (Switzerland)	2018	Waste management (value of materials)	Government, industry, academia
Aguiñaga E.; Henriques I.; Scheel C.; Scheel A	Building resilience: A self-sustainable community approach to the triple bottom line	Journal of Cleaner Production	2018	Waste management (residue, waste and by-product chains)	Community (households, farms, plantations, factories)
Velenturf A.P.M.; Purnell P	Resource recovery from waste: Restoring the balance between resource scarcity and waste overload	Sustainability (Switzerland)	2017	Waste management	Academia, industry, government
Pialot O.; Millet D.; Bisiaux J	“Upgradable PSS”: Clarifying a new concept of sustainable consumption/production based on upgradability	Journal of Cleaner Production	2017	Waste management (end of life option; upgradable product-service system, value chain)	Consumers & two manufacturers
Moggi S.; Dameri R.P	Circular business model evolution: Stakeholder matters for a self-sufficient ecosystem	Business Strategy and the Environment	2021	Innovative circular business model (surplus food redistribution)	RiCibo partnership in CE & local community
Shahbazi S.; Jönbrink A.K	Design guidelines to develop circular products: Action research on Nordic industry	Sustainability (Switzerland)	2020	Innovative circular business model (urban furniture, machines, equipment, tools)	Four companies
Pieroni M.P.P.; McAloone T.C.; Pigosso D.C.A	From theory to practice: Systematising and testing business model archetypes for circular economy	Resources, Conservation and Recycling	2020	Innovative circular business model (capital goods, furniture, electronics, clothing, medical devices)	Six manufacturing companies
Real M.; Lizarralde I.; Tyl B	Exploring local business model development for regional circular textile transition in France	Fashion Practice	2020	Innovative circular business model (textile & fashion)	Social enterprises
Pieroni M.P.P.; McAloone T.C.; Pigosso D.C.A	Configuring new business models for circular economy through product-service systems	Sustainability (Switzerland)	2019	Innovative circular business models (furniture sector)	Two manufacturing companies
Bocken N.M.P.; Schuit C.S.C.; Kraaijenhagen C	Experimenting with a circular business model: Lessons from eight cases	Environmental Innovation and Societal Transitions	2018	Innovative circular business model (consumer durables, clothing, agriculture, product sharing platform, water services)	Eight companies
Linder M.; Williander M	Circular business model innovation: Inherent uncertainties	Business Strategy and the Environment	2017	Innovative circular business models (bicycle manufacturing)	Researchers and company
Bocken N.M.P.; Miller K.; Weissbrod I.; Holgado M.; Evans S	Business model experimentation for circularity: Driving sustainability in a large international clothing retailer	Economics and Policy of Energy and the Environment	2017	Innovative circular business model (clothing)	Industry (large clothing retailers)

## Footnotes

1 TS = (circular economy) AND (TS = (business) OR TS = (economics) OR TS = (management) OR TS = (organization)). Refined by: DOCUMENT TYPES = (ARTICLE OR REVIEW ARTICLE) AND LANGUAGES = (ENGLISH).

2 Web of Science comprises 151 research areas, which represent a subject categorization scheme that is the same across all databases.

3 We provide a table of the top 20 journals with the most publications in CE research in the fields of business and economics in the appendix (Table 4).
